# Effects of organization and disorganization on pleasantness, calmness, and the frontal negativity in the event-related potential

**DOI:** 10.1371/journal.pone.0202726

**Published:** 2018-08-30

**Authors:** Sandra J. E. Langeslag

**Affiliations:** Department of Psychological Sciences, University of Missouri–St. Louis, St. Louis, MO, United States of America; Boston Children's Hospital / Harvard Medical School, UNITED STATES

## Abstract

A preference for organization is associated with several disorders, but is widespread in the general population as well. It remains unclear whether organization and various degrees of disorganization elicit pleasant or unpleasant feelings (i.e., valence), calming or arousing feelings (i.e., arousal), and a frontal negativity in the event-related potential (ERP) related to cognitive control. This study tested how organization, slight disorganization, and total disorganization affect valence, arousal, and the frontal negativity. Participants passively viewed organized, slightly disorganized, totally disorganized, and control pictures while their electroencephalogram was recorded. They also rated the valence and arousal elicited by each picture and completed questionnaires assessing desire for order and organization behavior. Organized pictures made participants feel most pleasant, control pictures made participants feel less pleasant, slightly disorganized pictures made participants feel even less pleasant, and totally disorganized pictures made participants feel least pleasant. There were no significant effects on arousal. Totally disorganized pictures elicited a frontal negativity in the ERP between 200–2000 ms after stimulus onset, which might reflect inhibition of rearranging behavior. Individual differences in desire for order and organization behavior did not correlate with valence, arousal, or the frontal negativity. The current study design and findings could be a starting point for examining the differences between adaptive and maladaptive preferences for organization and aversions to disorganization.

## Introduction

Does it make you happy when things are neatly organized? Does it annoy you when one item disturbs the neat organization? Does total disorganization make you feel uneasy? A preference for symmetry and order is associated with several disorders, such as obsessive-compulsive disorder and obsessive-compulsive personality disorder [1], Tourette syndrome [2], and autism spectrum disorder [3]. But a preference for organization is widespread in healthy people as well [4], probably because organization is associated with predictability [5]. Nevertheless, experimental research on the subjective experience of, and the brain’s response to organization and disorganization is scarce.

In an implicit association task, undergraduate student participants were faster and more accurate to categorize words related to organization (such as ‘symmetrical’ and ‘aligned’) with happy than disgusted faces, and words related to disorganization (such as ‘cluttered’ and ‘scattered’) with disgusted than happy faces [6]. This suggests that people link organization more with happiness than disgust, and disorganization more with disgust than happiness [6]. However, because perceiving a happy face is not necessarily associated with feeling happy and perceiving a disgusted face is not necessarily associated with feeling disgusted [7], this study does not show whether and how organization and disorganization influence subjective feelings. In another study [4], in contrast, participants with a strong preference for order felt more anxious after preparing a speech in a disorganized compared to an organized environment. In addition, undergraduate student participants indicated that they would feel more comfortable in an organized compared to a totally disorganized scene depicted on a picture, which was interpreted to indicate that organization is associated with feelings of calmness [4]. It is important to note that emotions are often classified on the two independent dimensions of valence and arousal, where valence refers to whether a feeling is pleasant or unpleasant, and arousal to whether a feeling is calming or exciting [8]. It therefore remains unclear whether feeling less anxious and more comfortable with organization than disorganization reflects a difference in valence (i.e., feeling more pleasant with organization than disorganization), a difference in arousal (i.e., feeling calmer with organization than disorganization), or both.

In a third study, healthy participants viewed pictures of organization and pictures of slight disorganization (e.g., a neat stack of slide frames with one frame out of line) and reported similar levels of distress in both conditions [2]. This suggests that slight disorganization does not affect how people feel, although the use of the rather strong term ‘distress’ could have resulted in a floor effect. Although it has been suggested previously to contrast total organization with slight disorganization [4], to my knowledge no study has compared subjective feelings in response to organization with both slight and total disorganization. In addition, because the previous studies did not include a control condition (i.e., a baseline condition that displayed items in their typical arrangement), it remains unknown whether organization is associated with an increase in pleasantness and/or calmness compared to baseline, whether disorganization is associated with a decrease in pleasantness and/or calmness compared to baseline, or both.

Therefore, the first goal of the current study is to determine how organization, slight disorganization, and total disorganization affect valence and arousal. For valence, it was expected that pictures of organization would elicit more pleasant feelings than control pictures, that pictures of slight disorganization would elicit more unpleasant feelings than control pictures, and that pictures of total disorganization would elicit more unpleasant feelings than both control and slight disorganization pictures. For arousal, it was expected that pictures of organization would elicit more calmness than control pictures, that pictures of slight disorganization would elicit more arousal than control pictures, and that pictures of total disorganization would elicit more arousal than both control and slight disorganization pictures.

Few studies have examined the brain’s response to organization and disorganization. In the above-mentioned study [2], healthy participants showed increased positron emission tomography (PET) activation in the visual cortex, motor cortex, and dorsal prefrontal cortex when looking at pictures of slight disorganization compared to pictures of organization. Because the prefrontal cortex is involved in cognitive control [9] and because prefrontal cortex activation was negatively correlated with the self-reported urge to rearrange the objects in the pictures, the prefrontal cortex activation in response to pictures of slight disorganization was interpreted as reflecting successful inhibition of rearranging behavior [2]. In an event-related potential (ERP) study, a figure depicting two lines that were almost parallel (i.e., slight disorganization) elicited a greater negativity over frontal electrodes between 270 and 600 ms after stimulus onset than a figure depicting two parallel lines (i.e., organization) [6]. This frontal negativity might be related to the N2 component of the ERP, which is a negative wave peaking between 200 and 350 ms after stimulus onset. The N2 consists of several subcomponents including a frontocentral component associated with novelty or mismatch from a perceptual template and a later frontocentral component associated with cognitive control [10]. Interpreting the frontal negativity in response to slight disorganization as reflecting increased cognitive control would be in line with the prefrontal cortex activation in response to slight disorganization from the above-mentioned study [2]. However, because the parallel lines stimulus was presented as a standard stimulus in an oddball task (appearing on 80% of the trials) and the non-parallel lines stimulus was presented as a deviant stimulus (appearing on 20% of the trials), and because the frontocentral N2 is larger for infrequent than frequent stimuli [10], it is unclear if the difference between the organization and disorganization conditions was due to differences in organization or in frequency. Therefore, the second goal of the current study is to determine how organization, slight disorganization, and total disorganization affect the frontal negativity in the ERP when all stimuli are presented with the same frequency. Assuming that the previously observed frontal negativity in response to slight disorganization compared to organization was not due to differences in frequency, it was expected that pictures of organization would elicit a smaller frontal negativity than control pictures, that pictures of slight disorganization would elicit a larger frontal negativity than control pictures, and that pictures of total disorganization would elicit a larger frontal negativity than both control and slight disorganization pictures. Finally, we administered questionnaires to test whether and how individual differences in the desire for order and organization behaviors are associated with valence, arousal, and the frontal negativity in response to pictures of organization and disorganization.

## Materials and methods

### Participants

Twenty-five students of the University of Missouri–St. Louis volunteered to participate. Two male participants were excluded because of excessive electroencephalography (EGG) artifacts (see below), so 23 participants (18–32 yrs, *M* = 20.7, 11 men) were included in the analyses. Inclusion criteria were normal or corrected-to-normal vision, no neurological or psychiatric disorders, and no use of medication known to affect the central nervous system, which were assessed by self-report over email prior to the testing session. Twenty-two participants were right-handed and one participants was left-handed, as determined by a hand preference questionnaire [11]. The study was approved by the institutional review board of the University of Missouri–St. Louis (approval number 921551–2) and participants provided written informed consent according to the Declaration of Helsinki at the start of the testing session. Participants were remunerated with course credit or $20.

### Stimuli

The stimuli were 24 sets of four pictures of common items (M&Ms, magazines, a desk, tools, apples, ballet dancers, a Rubik’s cube, towels, flowers, utensils, pencils, etc.). Most pictures were obtained from various Internet sources and some were taken for the purpose of this study. Each picture set consisted of an organized picture (e.g., M&Ms sorted by color), a slightly disorganized picture (e.g., M&Ms sorted by color with one M&M out of place), a totally disorganized picture (e.g., broken M&Ms), and a control picture that displayed items in their typical arrangement and served as a baseline condition (e.g., a regular bowl of M&Ms). So, the four conditions were matched for picture content. Four research assistants (21–39 yrs, *M* = 26.0, 2 men) who did not know anything about the study rated how (dis)organized the pictures were on a 1–9 scale, where 1 = very disorganized, 5 = neutral, and 9 = very organized. On average, the organized pictures were rated as 7.7 (*SD* = 0.8), the slightly disorganized pictures as 5.3 (*SD* = 1.2), the totally disorganized pictures as 3.0 (*SD* = 0.6), and the control pictures as 5.7 (*SD* = 0.6). These ratings confirmed that the four conditions were ordered as expected on a disorganization-organization continuum. The pictures had varying sizes, but mean picture width, height, and area did not differ significantly between conditions, all *F*s(3,93) < 1, *ns*. The pictures were presented in color on a black background.

### Procedure

First, participants completed the orderliness scale of the International Personality Item Pool (IPIP) [12], which measures the desire for order. Participants indicted how accurately 10 statements (e.g., “Love order and regularity”) described them on a 1–5 scale, where 1 = very inaccurate and 5 = very accurate. The score on this orderliness scale can range from 10 (low desire for order) to 50 (high desire for order) (Chronbach’s alpha in current sample = .87). Participants also completed the organization scale of the Behavioral Indicators of Conscientiousness (BIC) [13] to measure organization behaviors. Participant indicated how often they took part in each of 18 behaviors (e.g., “Make lists”) on a 1–5 scale, where 1 = never and 5 = often. The score on this organization scale can range from 18 (low organization behaviors) to 90 (high organization behaviors) (Chronbach’s alpha in current sample = .92).

Then, the electroencephalography (EEG) cap was attached and participants completed a passive viewing task. Each trial consisted of a fixation cross with jittered duration between 500–1000 ms, a picture for 2000 ms, and a blank screen for 1000 ms. Participants were instructed to pay attention to the pictures, to limit movements, and to try to blink during the blank screen only. Participants completed three practice trials with pictures that were not used in the main task. The main task consisted of two blocks of 48 trials each, for a total of 96 trials. Trial order was pseudorandom with the constraints that trials of the same condition appeared no more than two times in a row and that each block half contained six pictures of each condition. Each picture was presented only once.

**Fig 1:**
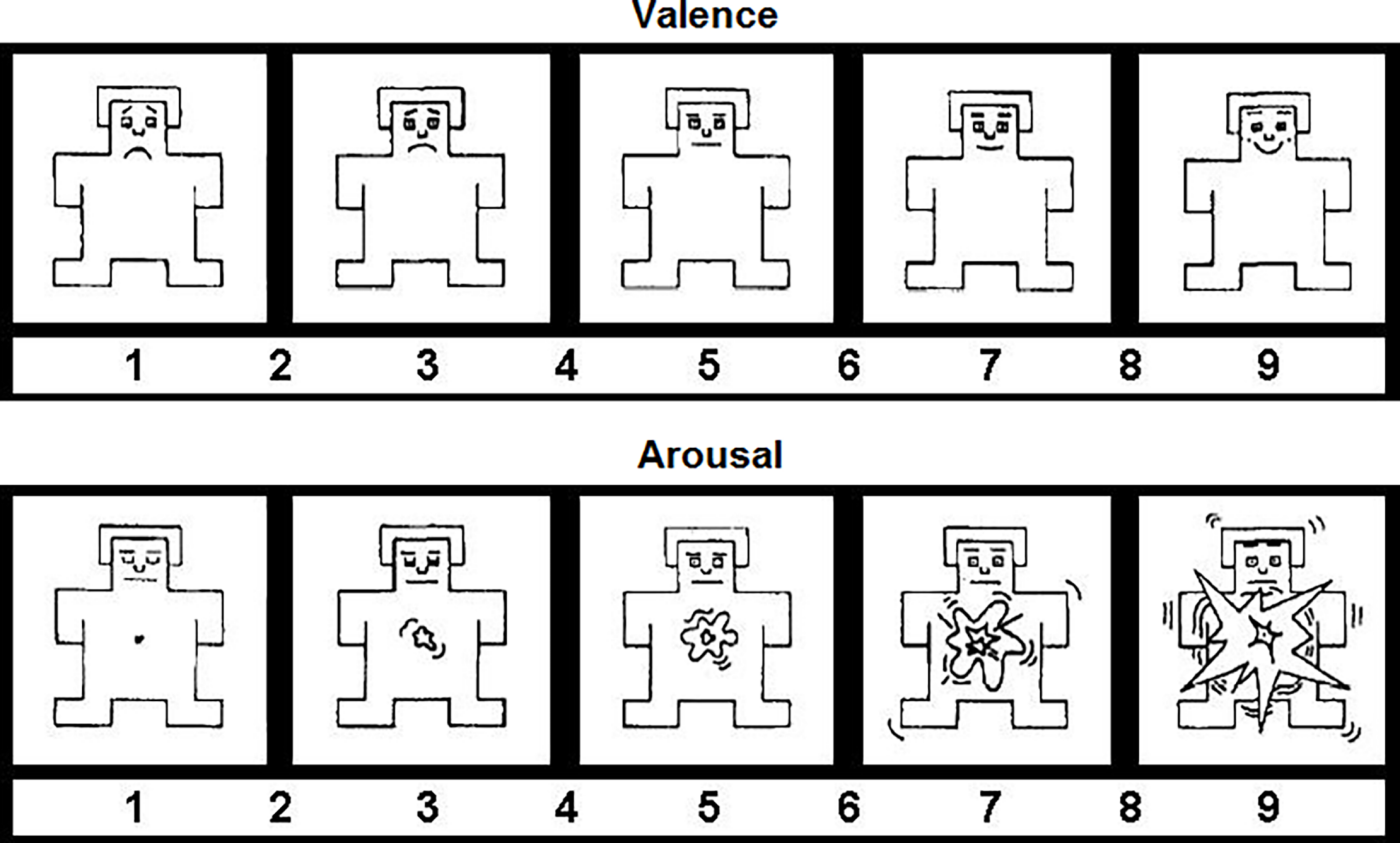
The valence and arousal rating scales of the Self-Assessment Manikin.

After completion of the passive viewing task, the electrode cap was removed and participants rated the valence and arousal of each picture with a computerized version of the Self-Assessment Manikin [14]. Specifically, participants were instructed to rate how unpleasant or pleasant and how calming or arousing each picture made them feel on a 1–9 scale, see Fig 1.

### EEG recording and signal processing

The EEG was recorded using a 32-channel amplifier and data acquisition software (ActiveTwo System, BioSemi). The 32 Ag-AgCl active electrodes were connected to the scalp through a head cap (BioSemi), according to the 10–20 International System (Fp1/2, AF3/4, Fz, F3/ 4, F7/8, FC1/2, FC5/6, Cz, C3/4, T7/8, CP1/2, CP5/6, Pz, P3/4, P7/8, PO3/4, Oz, O1/2). Vertical electrooculogram (VEOG) and horizontal electrooculogram (HEOG) were recorded by attaching additional electrodes (UltraFlat Active electrodes, BioSemi) above and below the left eye, and at the outer canthi of both eyes. Additionally, two electrodes were placed on the left and right mastoids (M1/2). An active electrode (CMS—common mode sense) and a passive electrode (DRL—driven right leg) were used to comprise a feedback loop for amplifier reference. Signals were digitized with a sampling rate of 512 Hz, a 24-bit A/D conversion, and a low pass filter of 134 Hz.

Data were analyzed with BrainVision Analyzer 2 (Brain Products, Gilching, Germany). A maximum of one bad electrode per participant was corrected using spherical spline topographic interpolation. Offline, an averaged mastoid reference was applied, because that is a common reference for the N2 [10]. The data were filtered using a 0.10–30 Hz band pass filter (phase shift-free Butterworth filters; 24 dB/octave slope) and a 60 Hz notch filter. The data were segmented in epochs from 200 ms before the onset of the picture until 2000 ms post-picture onset. Ocular artifact correction was applied semi-automatically according to Gratton, Coles, and Donchin [15]. The mean 200 ms pre-stimulus period was used for baseline correction. Artifact rejection was performed at individual electrodes with a baseline-to-peak minimum and maximum criterion of -75 to +75 μV. Two participants were excluded because they had fewer than half of the epochs left at one or more electrodes in one or more conditions after artifact rejection, leaving the previously reported number of 23 participants. In the included participants, the average number of accepted epochs per condition per electrode used in the analysis (see below) ranged from 22.7 to 23.6 out of 24.

### Statistical analyses

**Fig 2:**
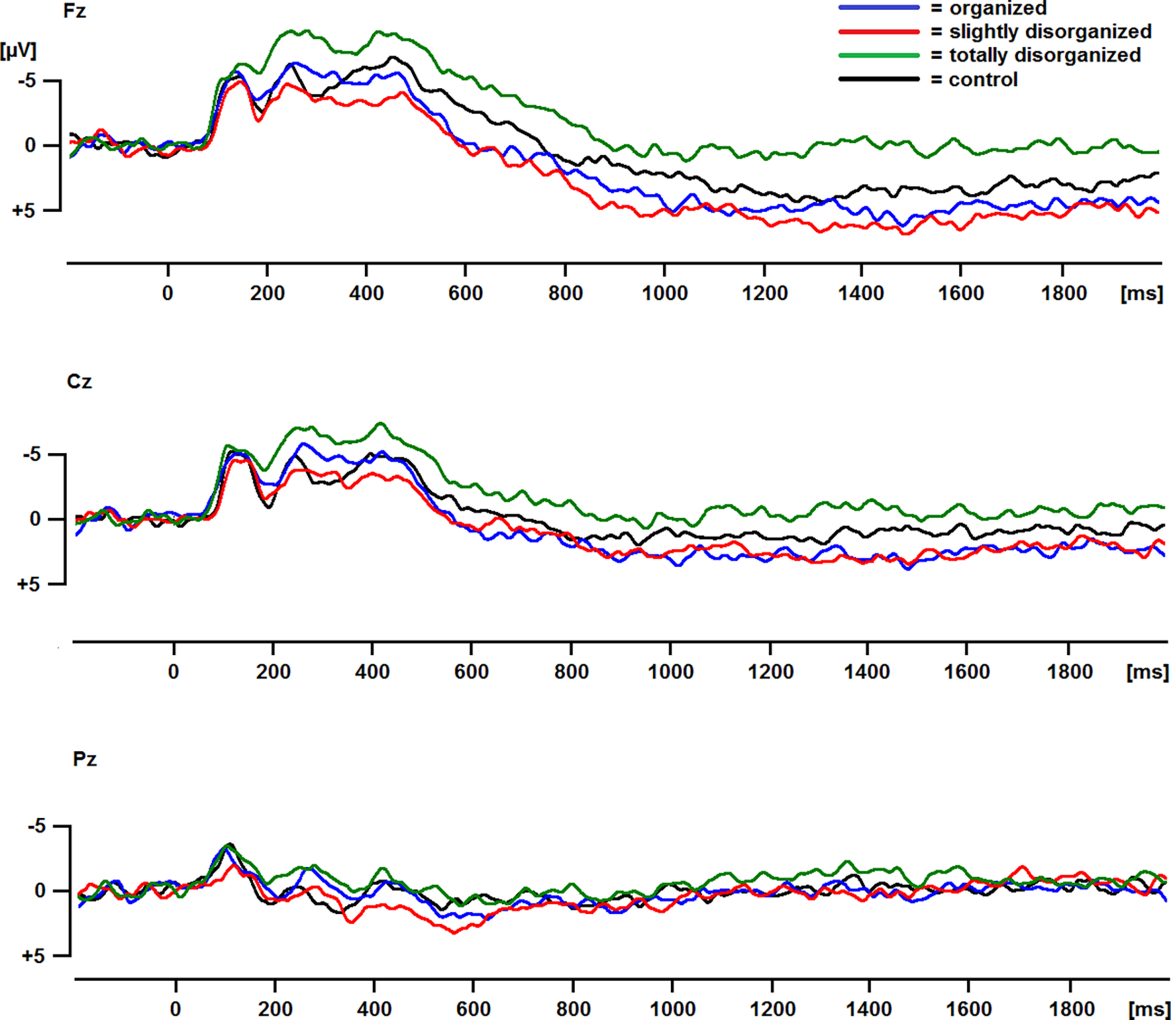
ERPs for each of the four conditions. Positive is plotted downwards.

After reverse scoring five of the IPIP orderliness items, the sum scores of the IPIP orderliness and BIC organization behavior scales were calculated. The valence and arousal ratings were analyzed with repeated measures analyses of variance (rmANOVAs) with the factor Picture (organized, slightly disorganized, totally disorganized, control). Visual inspection of the data showed that a frontal negativity emerged a little before 200 ms after stimulus onset, was maximal between 200–400 ms, and persisted until stimulus offset, see Fig 2. Therefore, the ERP was quantified by mean amplitudes in 200–1000 and 1000–2000 ms time windows at electrodes F3, Fz, F4, C3, Cz, C4, P3, Pz, and P4, which were subjected to rmANOVAs with factors Picture, Caudality (frontal, central, parietal), and Laterality (left, midline, right). Only significant effects involving the factor Picture are reported, because those are relevant for the research questions. When applicable, the degrees of freedom were corrected using the Greenhouse-Geisser correction. The *F* values, uncorrected degrees of freedom, the ε values and corrected *p* values are reported. An α level of 5% was selected. Significant main and interaction effects were followed-up using Holm’s procedure to control the type I error rate [16]. This procedure entails sorting the port hoc comparisons from smallest to largest *p* value. Then, the adjusted α level for each comparison is computed in this order by α / (*n*—*i* + 1), where α is 0.05, *n* is the number of comparisons, and *i* is the rank of the comparison. The *p* values are evaluated for significance against this adjusted α in rank order, and the procedure is terminated when a nonsignificant comparison is encountered.

Pearson correlations were computed between the valence and arousal ratings in each of the four conditions on the one hand, and the IPIP orderliness and BIC organization behavior scores on the other hand. Pearson correlations were also computed between the ERP amplitude averaged across electrodes F3, Fz, and F4 in each of the two time windows in response to each of the four conditions on the one hand, and the valence and arousal ratings in the corresponding conditions and the IPIP orderliness and BIC organization behavior scores on the other hand. See S1 Text for a comparison of participants with low and high levels of desire for order.

## Results

### Ratings

**Fig 3:**
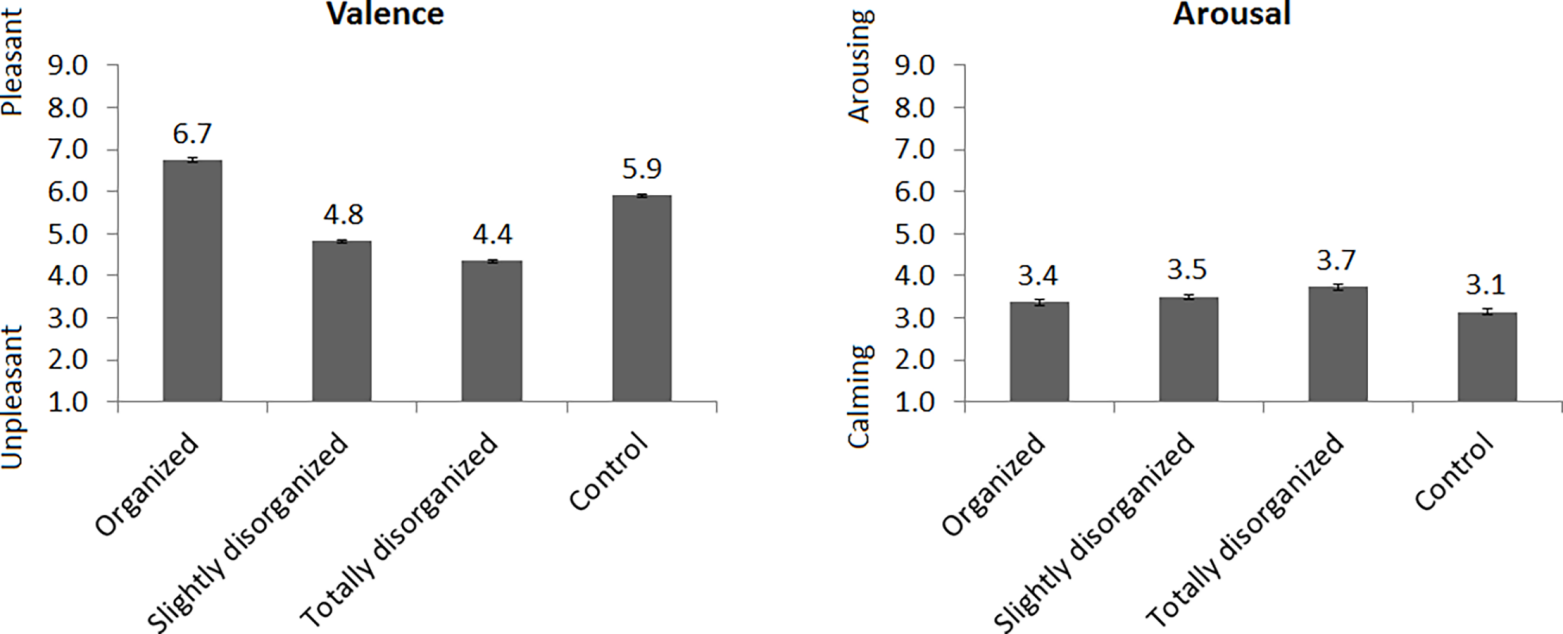
Valence and arousal ratings for each of the four conditions. Error bars indicate standard error of the mean.

See Fig 3 for the valence and arousal ratings. For valence, there was a main effect of Condition, *F*(3,66) = 69.1, ε = .61, *p* < .001. Follow-up tests showed that organized pictures were most pleasant, control pictures were less pleasant, slightly disorganized pictures were even less pleasant, and totally disorganized pictures were least pleasant, all *p*s < .009. For arousal, the main effect of Condition was not significant, *F*(3,66) = 2.4, ε = .54, *p* = .12.

### Event-related potentials

**Fig 4:**
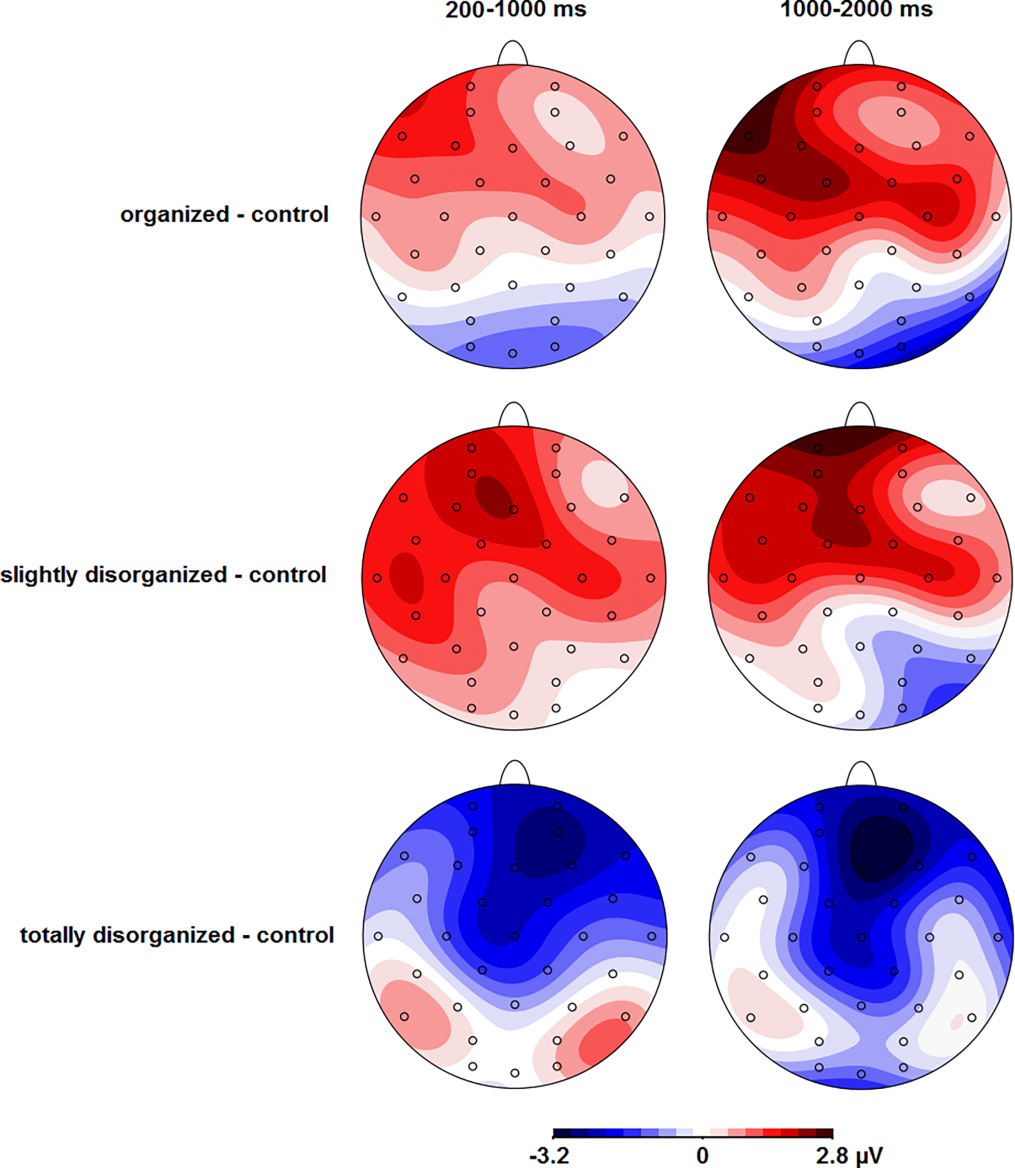
Scalp topographies of the difference waves in the two time windows.

See Fig 2 for the ERPs in response to the pictures in the four conditions and Fig 4 for the scalp topographies for the differences between the control and the other three conditions. Between 200–1000 ms, there was a main effect of Condition, *F*(3,66) = 6.9, ε = .92, *p* = .001, which was modulated by a significant Condition * Caudality interaction, *F*(6,132) = 7.9, ε = .66, *p* < .001. Follow-up tests showed that the ERP was more negative for totally disorganized than organized and slightly disorganized pictures at frontal and central electrodes, all *p*s < .002.

Between 1000–2000 ms, there was a main effect of Condition as well, *F*(3,66) = 3.9, ε = .80, *p* = .020, which was again modulated by a significant Condition * Caudality interaction, *F*(6,132) = 5.6, ε = .75, *p* < .001. Follow-up tests showed that the ERP was more negative for totally disorganized than organized and slightly disorganized pictures at frontal and central electrodes, all *p*s < .002.

### Individual differences

The average score on the IPIP orderliness scale was 33.0 (*SD* = 8.5, range = 15–50) and the average score on the BIC organization behavior scale was 51.9 (*SD* = 15.8, range 22–86). The ranges almost cover the possible ranges of each questionnaire, so participants varied considerably in their desire for order and organization behavior. The IPIP orderliness score and the BIC organization behavior score were positively correlated, *r*(21) = .58, *p* = .004, which confirms that the desire for order and organization behaviors are related.

The valence and arousal ratings in the four conditions did not correlate significantly with the IPIP orderliness or BIC organization behavior scores, -.27 < all *r*s(21) < .42, all *p*s > .052. The valence and arousal ratings in the four conditions also did not correlate with any of the corresponding ERP amplitudes at frontal electrodes, -.21 < all *r*s(21) < .27, all *p*s > .21. Finally, the ERP amplitudes at frontal electrodes in the two time windows in the four conditions did not correlate with the IPIP orderliness or BIC organization behavior scores, -.10 < all *r*s(21) < .33, all *p*s > .13.

## Discussion

The goal of this study was to determine how organization, slight disorganization, and total disorganization affect valence (i.e., how pleasant or unpleasant someone feels), arousal (i.e., how calm or aroused someone feels), and the frontal negativity in the ERP in healthy participants. To this end, participants viewed organized, slightly disorganized, totally disorganized, and control pictures that displayed items in their typical arrangement, while their EEG was recorded.

Valence ratings were modulated by organization and disorganization in accordance with the hypothesis. Compared to control pictures, organized pictures made people feel more pleasant, slightly disorganized pictures made people feel more unpleasant, and totally disorganized pictures made people feel most unpleasant. This is line with the previous finding that people reported to feel more comfortable with organization than total disorganization [4]. The inclusion of a control condition, however, extends that previous finding by showing that organization is associated with an increase in pleasantness compared to baseline and that disorganization is associated with a decrease in pleasantness compared to baseline. In addition, previous studies only included a slight disorganization condition [2] or a total disorganization condition [4], rather than both. The inclusion of both slight and total disorganization conditions in the current study reveals that total disorganization is more unpleasant than slight disorganization, which suggests an inverse relationship between the level of disorganization and pleasantness.

Unexpectedly, arousal ratings were not modulated by organization or disorganization. This implies that organization and disorganization do not impact how calm or excited people feel, which is in contrast to the previous suggestion that organization is associated with calmness [4]. That suggestion, however, was based on interpreting ‘comfortable’ as calming rather than the actual self-report of arousal. In the current study, the inclusion of both valence and arousal ratings and the observed dissociation between the two leads to the conclusion that organization and disorganization affect pleasantness, but not calmness.

Participants varied considerably in the personality trait orderliness, or desire for order, and in organization behavior. Nevertheless, the effects of organization and disorganization on valence were highly significant at the group level and the valence and arousal ratings did not correlate significantly with individual differences in desire for order and organization behavior. This supports the notion that a preference for order is widespread in the general population [4].

Totally disorganized pictures elicited a larger frontal negativity between 200–2000 ms after stimulus onset compared to pictures of organization and slight disorganization with control pictures non-significantly different in-between, which mostly aligns with the hypothesis. This frontal negativity was maximal between 200–400 ms, which corresponds with the typical latency of the N2 component of the ERP [10]. The observed frontal negativity for total disorganization is in line with the previous finding that two lines that were almost parallel (i.e., slight disorganization) and presented infrequently elicited a greater negativity over frontal electrodes between 270 and 600 ms after stimulus onset than two parallel lines (i.e., organization) that were presented frequently [6]. Because the totally disorganized pictures were presented with the same frequency as the pictures of the other conditions in the current study, the current finding shows that the frontal negativity in response to total disorganization is due to disorganization rather than to infrequent presentation. The frontal negativity in response to disorganized pictures did not correlate with valence or arousal ratings, or with desire for order or organization behavior, which suggests that the frontal negativity in response to disorganization is widespread in the general population. The N2 consists of several subcomponents including a frontocentral component associated with novelty or mismatch from a perceptual template and a later frontocentral component associated with cognitive control [10]. Total disorganization could be associated with novelty or a mismatch from a perceptual template because it may be unusual for common items such as the ones depicted in the pictures (M&Ms, magazines, a desk, tools, apples, ballet dancers, a Rubik’s cube, towels, flowers, utensils, pencils, etc.) to occur in a totally disorganized arrangement. In addition, total disorganization may be associated with cognitive control as it might require inhibition of the urge to rearrange the items into a more organized arrangement. Interpreting the frontal negativity in response to total disorganization as reflecting increased cognitive control would be in line with the previous findings that activation of the prefrontal cortex, which is involved in cognitive control [9], occurred in response to slight disorganization and was correlated with the self-reported urge to rearrange the objects in the pictures [2]. Future research could test if the frontal negativity in response to total disorganization correlates with the urge to rearrange or with measures of cognitive control including response inhibition. Future research could also test if the frontal negativity in response to total disorganization would be reduced if reorganization is less feasible, such as with natural scenes. Because the current results show that organization and disorganization affect the valence of subjective experience, future studies could also test whether organization and disorganization affect other ERP components that are sensitive to emotion, such as the early posterior negativity (EPN) and late positive potential (LPP) [17, 18].

In contrast to the hypothesis, slightly disorganized pictures did not elicit a larger frontal negativity than control pictures. In addition, the hypothesized smaller frontal negativity for organized compared to control pictures was not significant. These findings suggest that organized, slightly disorganized, and control conditions did not differ in novelty, perceptual mismatch from a perceptual template, and/or cognitive control.

A limitation of the current study is the relatively low number of participants, especially for the analysis of individual differences. The smaller sample and resulting lower power could explain the absence of significant correlations between valence and arousal ratings, questionnaire scores, and frontal negativity. It would be good to examine individual differences in future studies using larger samples. Another limitation is that even though the sample consisted of similar numbers of men and women, it was not large enough to test for gender differences. Because one study has shown a preference for symmetry in males but not in females [19], it would be interesting to study gender differences in valence, arousal, and the frontal negativity in response to organization and disorganization in future studies. A final limitation is the use of self-report measures for valence and arousal. It is important to realize though that self-report is the only way to assess how someone feels [20] since a feeling is the conscious awareness of an emotion [21]. A strength of the current study is the experimental manipulation of organization by including stimuli of organization, both slight and total disorganization, as well as control stimuli that displayed items in their typical arrangement and served as a baseline condition. Future studies could perhaps include a slightly organized condition that is orthogonal to slight disorganization (e.g., a regular bowl of M&Ms with a portion of it sorted by color) to distinguish between effects of slight disorganization and slight organization.

The stimuli were color pictures displaying common items against naturalistic backgrounds. The pictures of the four different conditions were carefully matched for picture content and size. However, because of the naturalistic nature of the pictures, it could be that the pictures of the different conditions varied in terms of low level visual characteristics, such as spatial frequency and visual complexity. Specifically, organization may be associated with lower spatial frequencies and visual complexities compared to control, whereas disorganization may be associated with higher spatial frequencies and visual complexity compared to control. Rather than confounding factors, however, spatial frequency and visual complexity may actually be defining features of organization and disorganization.

To conclude, this study examined the subjective experience of organization and disorganization in terms of valence and arousal, as well as the brain’s response to organization and disorganization. Organization increased pleasantness and disorganization decreased pleasantness, but organization and disorganization did not modulate calmness. In addition, total disorganization elicited a sustained frontal negativity in the ERP, which was tentatively interpreted as reflecting cognitive control. After confirming this interpretation in future research, subsequent studies could examine whether being in a disorganized environment uses up processing resources, just like worrying for example [22]. In addition, future research could focus on differences between healthy participants and patients (e.g., with obsessive-compulsive (personality) disorder, Tourette syndrome, and autism spectrum disorder) in the effects of organization and disorganization on valence, arousal, and brain function. Perhaps the effects of organization and/or disorganization on pleasantness are more extreme in patients than in healthy people. Or perhaps organization and/or disorganization do have an effect on arousal in patients, in contrast to healthy people. Finally, patients may show an abnormal brain response to organization and/or disorganization. For example, it has been shown that patients with Tourette syndrome had more activation in the anterior cingulate cortex and less activation in the visual, motor, and prefrontal cortices than healthy controls in response to slight disorganization compared to organization [2]. So it would be interesting to test whether the frontal negativity in response to disorganization differs between patients and healthy people. Comparisons like these would reveal the similarities and differences between adaptive and maladaptive preferences for organization and aversions of disorganization, which in turn could inform treatment. For example, perhaps emotion regulation, which is a core component of Cognitive-Behavioral Therapy and Acceptance and Commitment Therapy [23], could help patients change any abnormal emotional responses to organization and/or disorganization. Likewise, perhaps cognitive control training could help patients decrease any maladaptive organization behavior.

## Supporting information

S1 Text Comparison of participants with low and high levels of desire for order. (DOCX)
