# Supplementary material for: Effects of organization and disorganization on pleasantness, calmness, and the frontal negativity in the event-related potential
Source: PLoS One. 2018 Aug 30;13(8):e0202726. doi: 10.1371/journal.pone.0202726 (PMC6116999; doi:10.1371/journal.pone.0202726)
Supplement: S1 Text — (DOCX) [file pone.0202726.s001.docx]

**S1 Supporting Information. Comparison of participants with low and high levels of desire for order.**

Because mental disorders, including obsessive-compulsive (personality) disorder, were exclusion criteria, none of the participants had clinically dysfunctional levels of desire for order. Nevertheless, some participants did have very high levels of desire for order. To examine whether participants with very high levels of desire for order differed from the other participants in their responses to organized and slightly disorganized stimuli, I qualitatively compared the participants who scored below (*n* = 18) and above (*n* = 5) the 80^th^ percentile on the IPIP orderliness scale. The patterns of valence and arousal ratings and the frontal negativity between 200-1000 ms were similar between the two groups. Specifically, both groups numerically rated organized pictures as more pleasant (M_below_ = 6.9 and M_above_ = 6.2) than slightly disorganized pictures (M_below_ = 5.0 and M_above_ = 4.4). Similarly, both groups numerically rated organized pictures as less arousing (M_below_ = 3.5 and M_above_ = 2.9) than slightly disorganized pictures (M_below_ = 3.6 and M_above_ = 3.2). Likewise, the frontal negativity between 200-1000 ms was numerically greater for organized (M_below_ = -1.1 and M_above_ = -1.1) than slightly disorganized (M_below_ = -0.5 and M_above_ = -0.9) pictures in both groups. However, the results for the frontal negativity between 1000-2000 ms differed between the two groups. That is, the frontal negativity between 1000-2000 ms was numerically greater for organized (M_below_ = 3.9) than slightly disorganized (M_below_ = 4.0) pictures in the participants who scored below the 80^th^ percentile, whereas the frontal negativity was numerically greater for slightly disorganized (M_above_ = 6.1) than organized (M_above_ = 7.0) pictures in the participants who scored above the 80^th^ percentile. Interpreting the frontal negativity as reflecting cognitive control (10), this suggests that people with a very high desire for organization employ more sustained cognitive control when confronted with slight disorganization compared to organization than people with lower levels of desire for order.
